# Neurorehabilitation in dystonia: a holistic perspective

**DOI:** 10.1007/s00702-020-02265-0

**Published:** 2020-10-24

**Authors:** Lynley V. Bradnam, Rebecca M. Meiring, Melani Boyce, Alana McCambridge

**Affiliations:** 1 Department of Exercise Sciences, Faculty of Science, University of Auckland, Auckland, New Zealand; 2 Graduate School of Health, Discipline of Physiotherapy, University of Technology, Sydney, NSW Australia; 3 Department of Physiotherapy, Westmead Hospital, Sydney, NSW Australia

**Keywords:** Dystonia, Holistic, Rehabilitation

## Abstract

Rehabilitation for isolated forms of dystonia, such as cervical or focal hand dystonia, is usually targeted towards the affected body part and focuses on sensorimotor control and motor retraining of affected muscles. Recent evidence, has revealed people who live with dystonia experience a range of functional and non-motor deficits that reduce engagement in daily activities and health-related quality of life, which should be addressed with therapeutic interventions. These findings support the need for a holistic approach to the rehabilitation of dystonia, where assessment and treatments involve non-motor signs and symptoms, and not just the dystonic body part. Most studies have investigated Cervical Dystonia, and in this population, it is evident there is reduced postural control and walking speed, high fear of falling and actual falls, visual compensation for the impaired neck posture, and a myriad of non-motor symptoms including pain, fatigue, sleep disorders and anxiety and depression. In other populations of dystonia, there is also emerging evidence of falls and reduced vision-related quality of life, along with the inability to participate in physical activity due to worsening of dystonic symptoms during or after exercise. A holistic approach to dystonia would support the management of a wide range of symptoms and signs, that if properly addressed could meaningfully reduce disability and improve quality of life in people living with dystonia.

## Introduction

Dystonia is a neurological movement disorder, where one or more body parts are affected by involuntary, sustained or intermittent muscle contractions causing abnormal postures, repetitive movements, tics or tremors (Albanese et al. 2013). Cervical dystonia (CD) is the most common form of idiopathic isolated dystonia affecting the head and neck (Dauer et al. 1998), with motor dysfunction and pain causing significant distress and disability (Pauw et al. 2017; Dool et al. 2016). The most common medical treatment for CD are regular botulinum toxin injections (BTX) (Ferreira et al. 2007), even though patients express limited satisfaction (Comella and Bhatia 2015). Rehabilitation by allied health professionals usually takes the form of exercises of the neck, to reduce activity in contracted muscles and enhance strength and function of their antagonist muscles (Boyce et al. 2013; Pauw et al. 2014), with limited success. Many studies have provided physiotherapy with and without BTX in small clinical trials with varying results (Counsell et al. 2016; Hu et al. 2019; Tassorelli et al. 2006; Dool et al. 2019). There have been different rehabilitation approaches for CD, (Prudente 2018; Delnooz et al. 2009), however, these interventions all pirmarily target the impaired musculature of the neck. While motor symptoms such as head tremor, abnormal head posture and jerks are among the most burdensome symptoms, there is emerging evidence of non-motor symptoms such as pain, fatigue, sleep and mood disorders that further limit daily life functions and negatively impact on quality of life (Smit et al. 2016, 2017a, b). Recent studies showing deficits in balance, gait and function (Barr et al. 2017), indicate a more holistic approach to rehabilitation is needed (Batla et al. 2019). In the current review, relevant dysfunctions outside the dystonic body part/s and their importance for holistic rehabilitation are summarised.

## Physical function, gait and balance

**Fig. 1:**
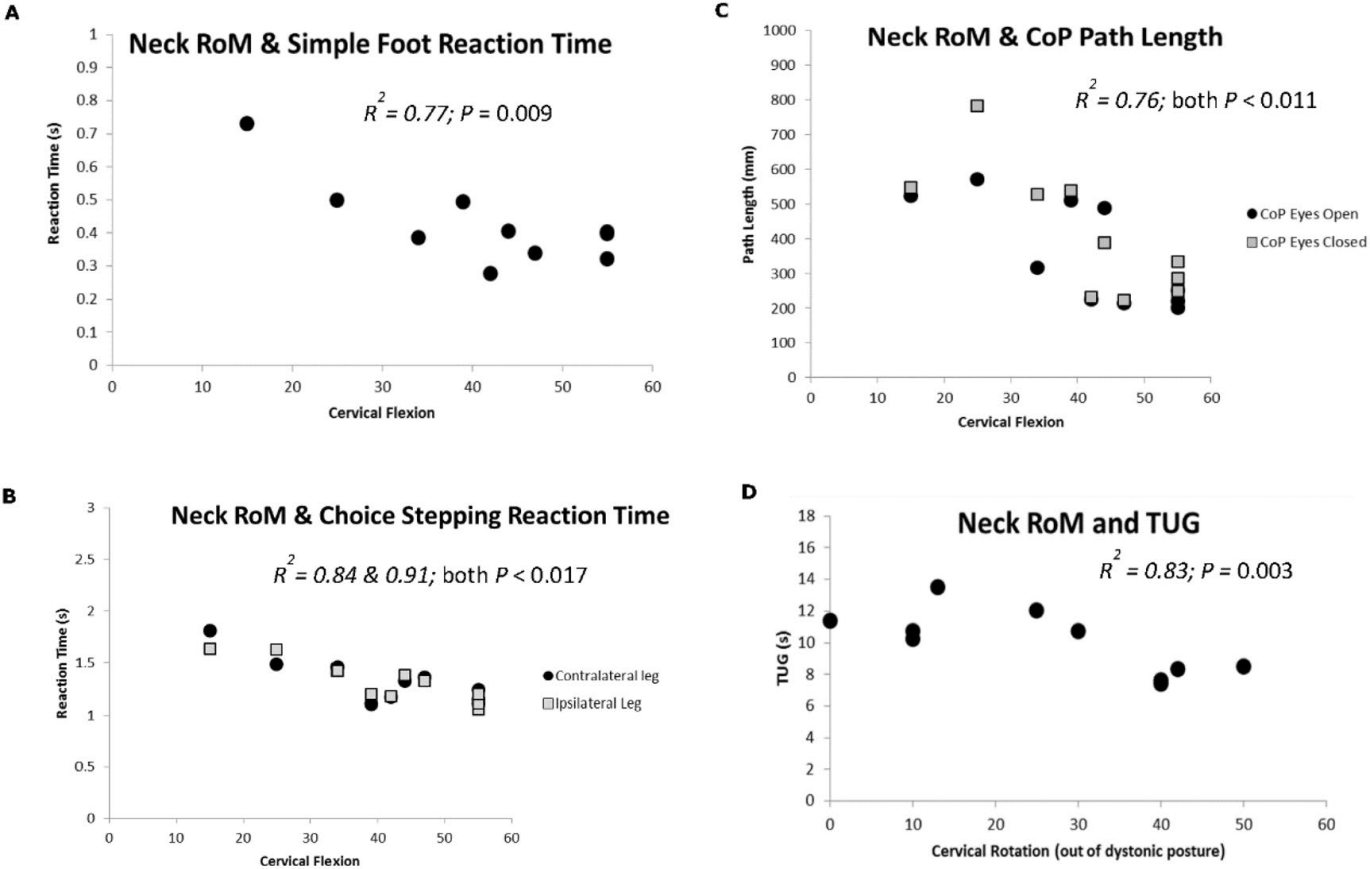
Correlations between cervical range of motion and functional measures from the study by Barr et al. (2017). **a** Cervical flexion and simple foot reaction time (RT), demonstrating longer RT was associated with reduced cervical flexion range of motion. **b** Cervical flexion and choice foot reaction time, demonstrating longer RT for both feet (ipsilateral and contralateral to the head turn direction) was associated with reduced cervical flexion. **c** Cervical flexion and COP path length during force plate postural sway measures for eyes open and eyes closed conditions, demonstrating greater COP pathlength for both conditions were associated with reduced cervical flexion. D. Cervical rotation (contralateral to dystonic rotation) and timed-up-and-go (TUG) test, where longer TUG times were associated with reduced cervical rotation

**Fig. 2:**
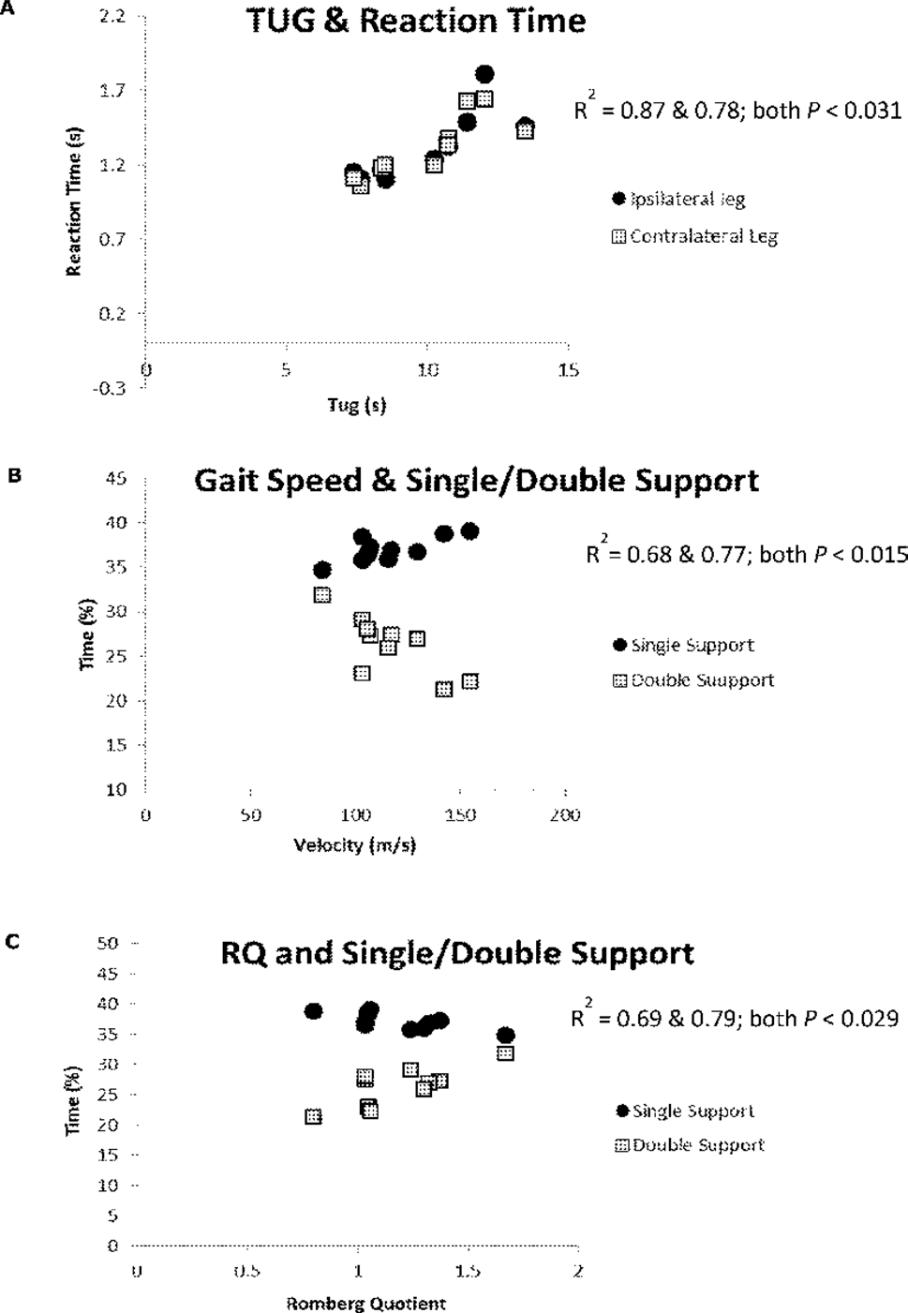
Correlations between functional measures from the study by Barr et al. (2017). **a** TUG and choice stepping reaction time, where longer RT was associated with slower times to complete the TUG. **b** Walking speed and time spend in single and double support, where longer time in double support and shorter time in single support was associated with slower walking speed. **c** Time spend in single and double support and the rhomboid quotient (RQ), where longer time in double support and shorter time in single support was associated with a lower RQ. Lower RQ indicates greater sway with eyes closed relative to eyes open, hence this finding indicates those with more ‘normal’ RQ’s actually presented with the impaired gait pattern

People living with CD exhibit impairments in physical function; not surprising considering the vital role of proprioception, visual and vestibular feedback in maintaining upright posture and balance. Central nervous system (CNS) processing of sensory inputs from all three systems are impacted by the head turn posture. Consequently, assessment of postural control and gait function should be included in physical assessments of dystonia patients. Emerging evidence demonstrates gait deficits and slower walking speed in people with CD when spatiotemporal parameters are measured in a laboratory setting (Barr et al. 2017; Hoffland et al. 2014; Esposito et al. 2017). Ten people living with CD were compared to ten control adults across a range of gait kinematic measures assessed using an instrumented walkway (Barr 2017). The dystonia group on average displayed reduced step length and increased step time, and spent more time in double support (both feet on the ground), all of which are consequences of poor balance control. In support, the time to perform the timed-up-and-go (TUG) test, a common test of physical function involving gait speed, turning and balance, was increased in CD (Barr et al. (2017)). The CD group demonstrated greater postural sway, as measured by centre of pressure (COP) path length using a force plate, and increased choice, but not simple stepping reaction time. The functional deficits may be related to the aberrant head posture, as reduced cervical range of motion (neck flexion) was significantly correlated to delayed simple and choice reaction times and increased postural sway, while limited cervical rotation was associated with slower TUG completion time (Fig. 1a–d). There were also strong correlations between functional measures, with a longer time to complete the TUG associated with greater delay in stepping reaction times (Fig. 2a). Longer time spend in double support and reduced time in single support during gait is a known strategy for enhancing stability during walking. Not surprisingly, there was correlation between these measures in people living with CD (Fig. 2b), indicating maintaining balance during gait may be an issue for some, slowing down walking speed. Finally, longer time spend in double support and reduced time in single support was also associated with a higher rhomboid quotient (RQ) (Fig. 2c). The RQ is a ratio of postural sway between eyes closed and eyes open, and as normal adults sway more with eyes closed, the RQ is usually between 1 and 2. However, CD patients had lower RQ’s than controls as they swayed more with their eyes open in comparison to eyes closed. The RQ in dystonia was also correlated with gait kinematics (Barr et al. 2017). Greater postural sway amplitude and velocity was also reported in people living with CD compared to control adults during sitting postures; particularly noticeable in those with head tremor and more impaired cervical sensorimotor control (Pauw et al. 2018). We recently investigated postural sway in ten people with CD and ten matched controls, using an accelerometer attached to the waist rather than the gold-standard force plate. We recorded root mean square (RMS) of the acceleration in medial–lateral and anterior–posterior directions during eyes open and eyes closed conditions while in tandem stance. We found greater acceleration in the medial–lateral but not anterior–posterior directions in the CD group, and this postural sway was increased in the presence of head tremor. Instability in the medial–lateral direction could be an important new finding, as it can differentiate fallers from non-fallers in community-dwelling older adults (Park et al. 2014), so has potential as a screening test for falls risk in dystonia. Current treatment of CD is focused on the cervical region, however, the evidence summarised highlights the value of adding physical function assessments, and postural control and/or stepping reaction exercises along with gait rehabilitation into the therapeutic management of dystonia.

## Falling and fear of falling

Impairments of gait, balance and vision may impact on people with dystonia in ways that could result in falls. Fear of falling is high in people with CD, and was first reported by Hoffland and colleagues (Hoffland et al. 2014) using the Activities-Specific Balance Confidence Scale (ABC), and by Barr and co-workers (Barr et al. 2017) using the Falls Self-Efficacy Scale-International (FES-I). We later conducted an international survey of a mixed dystonic population to further explore the falls experience, and found 39% of the 122 respondents reported falling over in the previous 6 months (Boyce et al. 2017, 2020). Many of the fallers were living with isolated forms of dystonia such as CD, blepharospasm and focal hand dystonia, and not dystonia directly affecting the trunk and/or lower limbs. This suggests falling may be a consequence of the physical function impacts of dystonia, such as poor sensorimotor control, balance and gait function. In that study, fallers had significantly more fear of falling and poorer balance confidence than those who did not fall according to the ABC and FES-I scales (Boyce et al. 2017, 2020). We then validated both scales in the dystonia population and determined cut off points indicating falls risk for each scale. The cut off points for the ABC scale was 71.3 out of 100 (Boyce et al. 2017), somewhat lower than scores reported in Parkinson’s disease (Hoehn and Yahr 3, ABC cut off = 81) (Bello-Haas et al. 2011) and Multiple Sclerosis (mean score = 79) (Cameron and Huisinga 2013), but similar to the falls cut-off score of 67 reported in the healthy elderly (Lajoie and Gallagher 2004), despite the relatively younger sample of dystonia participants that completed our survey (51.2 ± 21.1 years). Similarly, the FES-I cut off point for a fall in the dystonia population was 29.5 out of 64 which is close to that of 29.6 reported in a mixed neurological population (Jonasson et al. 2014) and within the range that defines “high risk of falls” (cut off score > 23) in the healthy elderly (Delbaere et al. 2010). From the current research, it appears that people with dystonia report less fear of falling and higher balance confidence than people with other progressive neurological diseases, but similar fear of falling and balance confidence to older healthy people. This points to the importance of assessing balance and falls risk in the dystonia population during rehabilitation, even in those with isolated forms affecting the neck, face, voice or hand, and not just people with truncal or lower limb dystonia.

There were a variety of reasons for falling reported by survey respondents (Boyce et al. 2020, 2017). The most common reasons were related to losing balance when walking, turning, reaching or using stairs, as illustrated by the following quotations from study participants; “walking and just lost balance “; “loss of balance and my feet are turning inwards”; and “lost my balance standing and walking”. Falling after tripping was also common, and often related to restricted vision due to CD or blepharospasm (BLP), as per the participant quotes; “Tripped over a raised metal edging outside of a shopping centre”; “tripped when stepping out from a picnic shelter”; “tripping over carpet rugs”; and “I was walking over my farm and didn't see a small rabbit hole which my foot went into”. Falls were reported as occurring as a direct result of dystonic impairments, illustrated by the quotes of; “I often lose track of where I am on stairs and sometimes skip steps going down stairs and fall”; “shopping in stores, always bumping into displays, people and certain lighting set Dystonia off”; “both feet turned in and I was walking on my ankles which made me fall down”; and “had Blepharospasms lost balance, or something in the way I didn't see”. Falls may occur secondarily to impaired vison, balance or proprioception due to dystonia, or caused by dystonic impairments causing difficulty in scanning the environment for hazards to ambulation. Tripping over objects due to poor vision related to a fixed head posture in CD or eye closure in blepharospasm may also cause falls, rather than impaired balance per se. This is supported by our research showing reduced vision-related quality of life in dystonia (Bradnam et al. 2020), where participant quotes also spoke of tripping or falling due vision impairment secondary to dystonic postures (see below). We recently completed a prospective study in adults with CD, investigating functional balance and walking tests using scales validated in other neurological populations or healthy older people. Our preliminary analysis suggests balance and mobility may not be as impaired as previously indicated, as people with CD mostly performed well on the physical functional scales. Therefore, it is possible that the fear of falling previously reported (Boyce et al. 2020) may be more related to psychological rather than physical limitations. While this idea is speculative and requires further investigation, it has an important impact on the way people with dystonia are managed during rehabilitation following a fall. In these cases, improving balance confidence from a psychological perspective could be more influential than physical or exercise-based rehabilitation. Regardless, falls-related fear should be addressed from a multi-disciplinary perspective, and falls prevention in the most appropriate format based on robust physical and psychological assessment should be included in rehabilitation programs.

## Vision and function

People living with CD exhibit increased postural sway with their eyes open compared to control adults, indicating vision is not used to maintain centre of gravity within the base of support to the same degree as normal (Barr et al. 2017). This may arise due to the abnormal head posture in CD, meaning vision cannot be relied upon to provide reference points for spatial orientation and balance. Certainly, vision impairment as a consequence of dystonic postures is a major issue for many people living with CD. A survey of 42 mixed dystonia participants found reduced vision-related quality of life compared to normative data (Bradnam et al. 2020). People living with dystonia significantly differed on two domains of a vision-related quality of life questionnaire; ocular symptoms and role performance. The following participant quotes supported reduced vision-related quality of life in a powerful way; ‘blurriness, tired eyes, eyes not facing what I want to see due to twisted head—have to look out of the corner of my eye or not look at all’; ‘focusing difficulty, judgement of distance in regard to steps and narrow walkways. I become unbalanced easily’; ‘my field of vision is affected when walking by head pulling to right’; ‘the only difficulty I have is looking at things directly because my head turns. That is, I find I have to look at some things with my peripheral vision’; ‘because I need to assist my head to be straight forward with my hand is my main problem. When I do this I can see quite o.k.’. These previously unpublished quotes point to a relationship between head posture, vision and functional impairments, including balance, which may help to explain the incidence of falls and high fear of falling in the dystonia population outlined in the previous section. Neurological vision deficits should also be considered in CD, as we found one young female participant (out of ten) to have marked visuospatial neglect using a battery of computer-based and pencil and paper spatial neglect tests (Bradnam et al. 2020). Clinicians should be aware of the possibility their CD patients could experience neglect and screening for this should be included in a comprehensive assessment of dystonia. In this study, we used mobile eye-tracking to determine the direction of eye movements made by people with CD when navigating an indoor circuit and identifying visual targets. Participants made a significant number of eye movements away from the dystonic head turn direction, indicating compensatory behaviour arising from the abnormal head posture (Bradnam et al. 2020). What is unknown, and of potential important impact for navigation around real world environments, is whether such compensatory eye movements induce fatigue in the oculomotor system. A fatigue-inducing paradigm in healthy young adults impaired saccade velocity (speed of eye movement) due to central neural mechanisms (Connell et al. 2016, 2017a, b). With relevance for individuals with CD, making compensatory eye movements may produce oculomotor fatigue with potential safety consequences when navigating fast-paced, real-life environments requiring visual compensation for a rotated neck, such as crossing a busy road. Clinicians and patients alike should be made aware of the potential for oculomotor fatigue secondary to repetitive eye movements in the contralateral direction to head turn so they can make the necessary compensations. We are currently investigating eye movement adaptation in people with CD and have found in our preliminary data from nine participants that normal adaptation occurs in the contralateral, but not ipsilateral, direction relative to the dystonic head turn. This may indicate the compensatory movements in the contralateral direction described by Bradnam et al. (2019), may provide adequate stimulation to the cerebellar structures responsible for saccade adaptation. This would not be the case for the ipsilateral direction as the eyes are rarely moved that way since the head is already turned. If this novel finding is maintained in a larger participant group it suggests eye movement training in the ipsilateral direction may be a therapeutic intervention to possibly normalise cerebellar-mediated saccade adaptation and oculomotor function. Vision impairment secondary to dystonic postures and its impact on physical function, visual compensation and oculomotor fatigue along with potential neurological impairments like spatial neglect should be considered important components of holistic rehabilitation of dystonia.

## Non-motor symptoms

Many people living with dystonia experience non-motor symptoms contributing to disability and reducing participation in daily activities (Smit et al. 2017a; Stamelou et al. 2012; Torres and Rosales 2017), leading to the development of non-motor symptom scales for dystonia (Smit et al. 2017a; Klingelhoefer et al. 2019). Non-motor symptoms featured strongly when people with CD were asked about their most burdensome symptoms (Smit et al. 2017a) and the most prevalent experienced by people with idiopathic, isolated dystonia were pain, depression, anxiety, apathy, and impaired sleep (Smit et al. 2017a; Novaretti et al. 2019). Other non-motor symptoms such as fatigue, catastrophizing, sensorimotor disturbances, olfactory and visual problems have also been noted amongst others, and also impact negatively on quality of life (Zetterberg et al. 2009). Non-motor symptoms are important when considering the overall management of dystonia as they play a significant role in quality of life (Smit et al. 2017a; Torres and Rosales 2017; Tomic et al. 2016).

Pain is a prevalent and debilitating non-motor symptom in dystonia. In people with CD, pain is reported in 55–89% of people (Avenali et al. 2018). The prevalence of pain in other dystonia types is lower than CD, with studies reporting pain was a symptom in 30–40% of people with FHD or lower limb dystonia and only 3% in BLP primarily related to photophobia pain (Avenali et al. 2018). Despite being a common co-occurring symptom of dystonia, current knowledge of the mechanism of dystonic pain is incomplete and effective management strategies are largely insufficient (Avenali et al. 2018). Pain is very likely not only a consequence of having a sustained muscle contraction caused by dystonia but may primarily arise from abnormal neural processing. Better understanding how people with dystonia describe the pain they experience is the focus of our current research, which will be used to help inform appropriate therapeutic strategies. To date, pain is most commonly treated with BTX and medication (Marciniec et al. 2019; Siongco et al. 2020). Novel cerebellar neuromodulation techniques also show promise for reducing pain in CD when combined with physical therapy as an adjuvant to BTX injections (Bradnam et al. 2014, 2016). Evidence for oral medications for management of pain in CD or other focal dystonia’s is lacking (Avenali et al. 2018).

Neuropsychiatric features also commonly coexist with dystonia, be the case focal, segmental or generalized, idiopathic or heredodegenerative dystonia’s (Torres and Rosales 2017). These broadly include depression, anxiety, personality disorder, obsessive–compulsive disorder (Stamelou et al. 2012), as well as reduced self-efficacy, catastrophizing, fear of movement, and stigma (Zetterberg et al. 2012). Neuropsychiatric comorbidities have been found to be significantly higher than controls and are prevalent in all forms of idiopathic dystonia, though the prevalence is particularly high in CD (up to 90%) (Avenali et al. 2018). Interestingly, psychiatric features such as depression and anxiety precede the onset of motor symptoms for many people, suggesting they may be a primary feature of dystonia (Fabbrini et al. 2010; Wenzel et al. 1998). However, given the debilitating experience of pain and many other symptoms that co-occur with dystonia it is likely that a proportion of patients experience depression as a secondary feature of living with their dystonic symptoms. Although the relationship between dystonia severity and the severity of depression is unclear, studies in CD or segmental and generalized dystonia patients show that mood does improve with positive treatment effects (Stamelou et al. 2012; Torres and Rosales 2017).

Fatigue appears to be a major issue and occurs independently to psychological factors and quality of sleep (Smit et al. 2017b; Wagle Shukla et al. 2016). Fatigue emerged as a significant barrier to participation in exercise and physical activity in our own research (McCambridge et al. 2019), detailed below. Fatigue in dystonia is worthy of further exploration and holistic rehabilitation strategies should include fatigue management. Fatigue commonly is associated with sleep disturbances and disrupted sleep is also a non-motor symptom of dystonia, with an estimated prevalence between 44 and 70% in CD and BLP patients (Eichenseer et al. 2014; Paus et al. 2011). Studies on sleep in dystonia have found a reduction in sleep quality and efficiency, less time in REM (rapid eye movement) sleep, and more awakenings (Silvestri et al. 1990; Sforza et al. 1991). Impaired sleep quality or excessive daytime sleepiness is associated with depression and anxiety in dystonia (Smit et al. 2017b; Wagle Shukla et al. 2016; Paus et al. 2011), however, the association between impaired sleep and severity of dystonia is mixed. Sleep quality is also not improved by BTX injection (Eichenseer et al. 2014), though this has not been specifically explored. Non-motor symptoms contribute significantly to the disability experience of those living with dystonia, and must be addressed to take a truly holistic approach to therapeutic management and rehabilitation (Torres and Rosales 2017).

## Exercise and physical activity

**Fig. 3:**
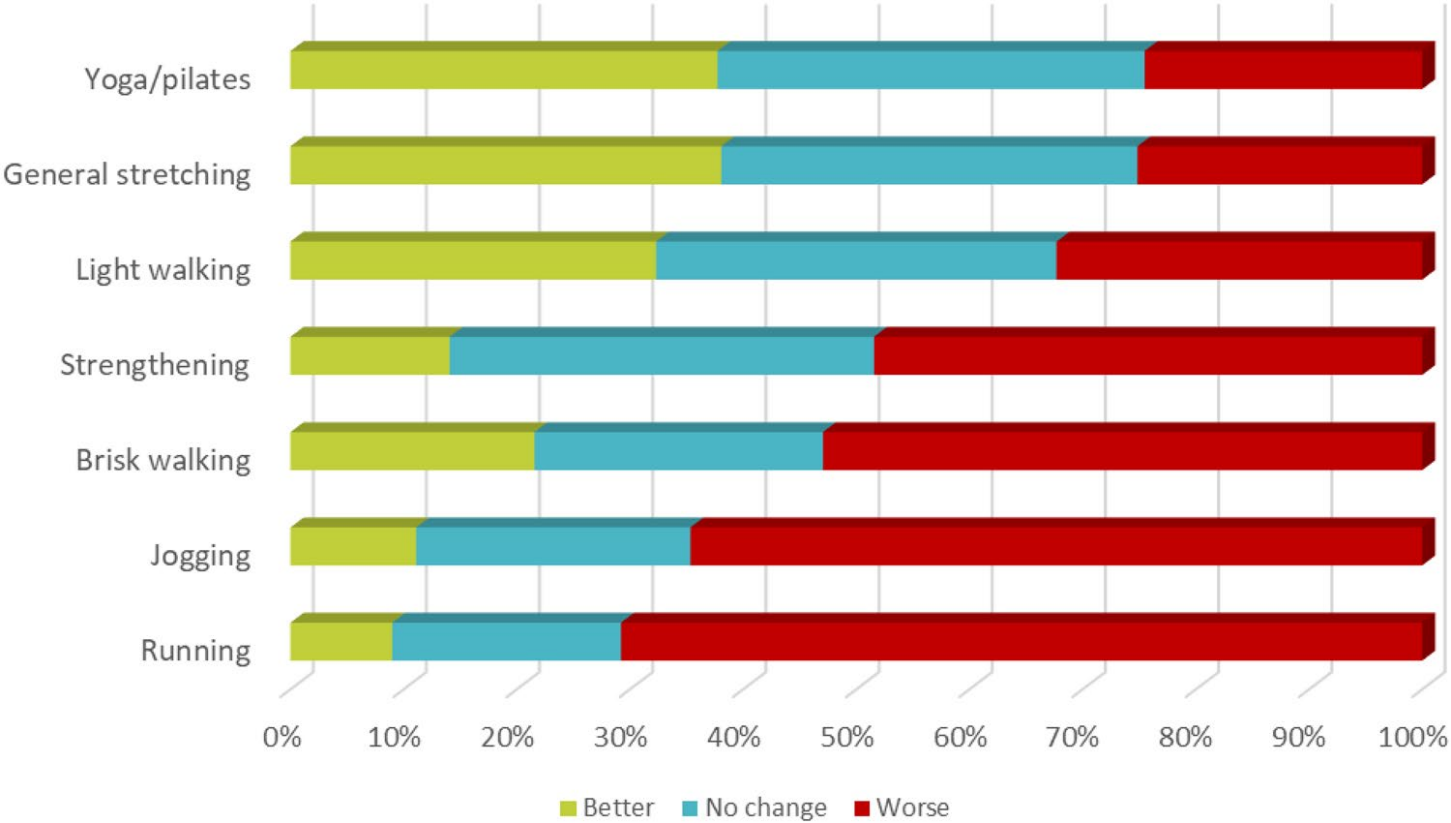
Physical activity and exercise and their impact on dystonia symptoms in survey respondents from the study by McCambridge et al. (2019) showing the proportion (percentage) of respondents that answered ‘better’ (green), ‘no change’ (blue) and ‘worse’ (red) for each activity. Obvious higher impact exercise tended to worsen dystonia, while low impact exercise may be beneficial, or at least not aggravating for around 2/3 of dystonia patients

Exercise is not only important for cardiometabolic health in general, but for neurological populations, it also has the potential to improve neuroplasticity and provide therapeutic benefits. All the above-mentioned movement impairments and non-motor symptoms experienced, along with vision-related impairments, have implications for the amount of physical activity (PA) and exercise that people living with dystonia engage in. Several neurological populations (Parkinson’s disease, Multiple Sclerosis, Stroke) have been extensively investigated for benefits of PA on disease-specific signs such as fatigue, depression and pain, and general cardiovascular and musculoskeletal health (Latimer-Cheung, et al. 2013a, b; Motl et al. 2018). In these conditions, remaining active can attenuate disease progression and physical deconditioning, and maintain or improve cognitive function, and exercise guidelines have been published (Kim et al. 2019). However, in a systematic review that analysed the number of studies assessing physical activity in neurological populations, no studies in people living with dystonia were included (Block et al. 2016). There is little understanding of how PA and exercise engagement may affect physical and psychological health in people living with dystonia. Even less research has investigated the effects that sedentary behaviour (SB), an independent risk factor for poor health, has on health in dystonic populations. Exercise guidelines specific to this patient cohort are needed. We recently published the first investigation of this kind; an international survey to determine the amount of PA and SB engagement and reported barriers and facilitators to physical activity using questionnaires (McCambridge et al. 2019). Upon analysis of the 263 people who completed the questionnaires, people living with dystonia appeared to achieve the minimum recommendations for PA, by means of incidental activity during transport and domestic activities (McCambridge et al. 2019). However, on average, people reported spending in excess of 8 h per day in sedentary behaviour. Common barriers to engaging in PA that were identified were personal barriers, relating to physical impairments, and financial barriers and a lack of trained exercise specialists (McCambridge et al. 2019). The most reported dystonic symptom barriers were pain, fatigue and poor balance. Zetterberg and colleagues (Zetterberg et al. 2015) found that employment as well as self-efficacy for exercise had the greatest association with physical activity in their survey of over 350 people with cervical dystonia (Zetterberg et al. 2015). Furthermore, many people with dystonia do not exercise as it tends to aggravate a range of dystonic symptoms (McCambridge et al. 2019). However, our survey revealed that lower intensity exercise was less aggravating for dystonia symptoms than high-intensity exercise (Fig. 3), a finding that has potential implications for prescribing exercise in practice. When asked what would help them be more active, symptom reduction was a common reply, as illustrated by the following participant quotes; *I'm as physically active as symptoms permit on any given day. So only reduced symptoms could make me more active’* and *‘To be less tired from medications; to be spasm and pain free; to have better posture and balance for more than a few minutes at a time; to have more energy; to have daily enjoyable beneficial short sessions of exercises or gentle short walks with supportive people. Encouragement and inclusion are motivating for everyone’.* Other quotes included;*’More energy, less social anxiety’, ‘Some way to reduce the tremor and muscle spasm’, ‘If I had better balance and movement in my neck’, Less pain, able to walk without pain’, ‘Increased motivation as I am so fatigued’ and ‘I am as physically active as I am capable of being without significantly aggravating my symptoms and pain’.*

People with dystonia face extensive barriers to physical activity and exercise engagement and more effective tailored interventions are needed to reap the benefits of activity for overall health and well-being. Future studies are critical as they have the potential to inform behaviour change addressing barriers in order to promote feasible and beneficial activity behaviours in people with dystonia. It is also important to understand motivational aspects of why people living with CD continue to exercise despite worsening of dystonic symptoms to inform a behavioural intervention with a self-management focus, and a study is currently underway for this.

## Discussion

**Fig. 4:**
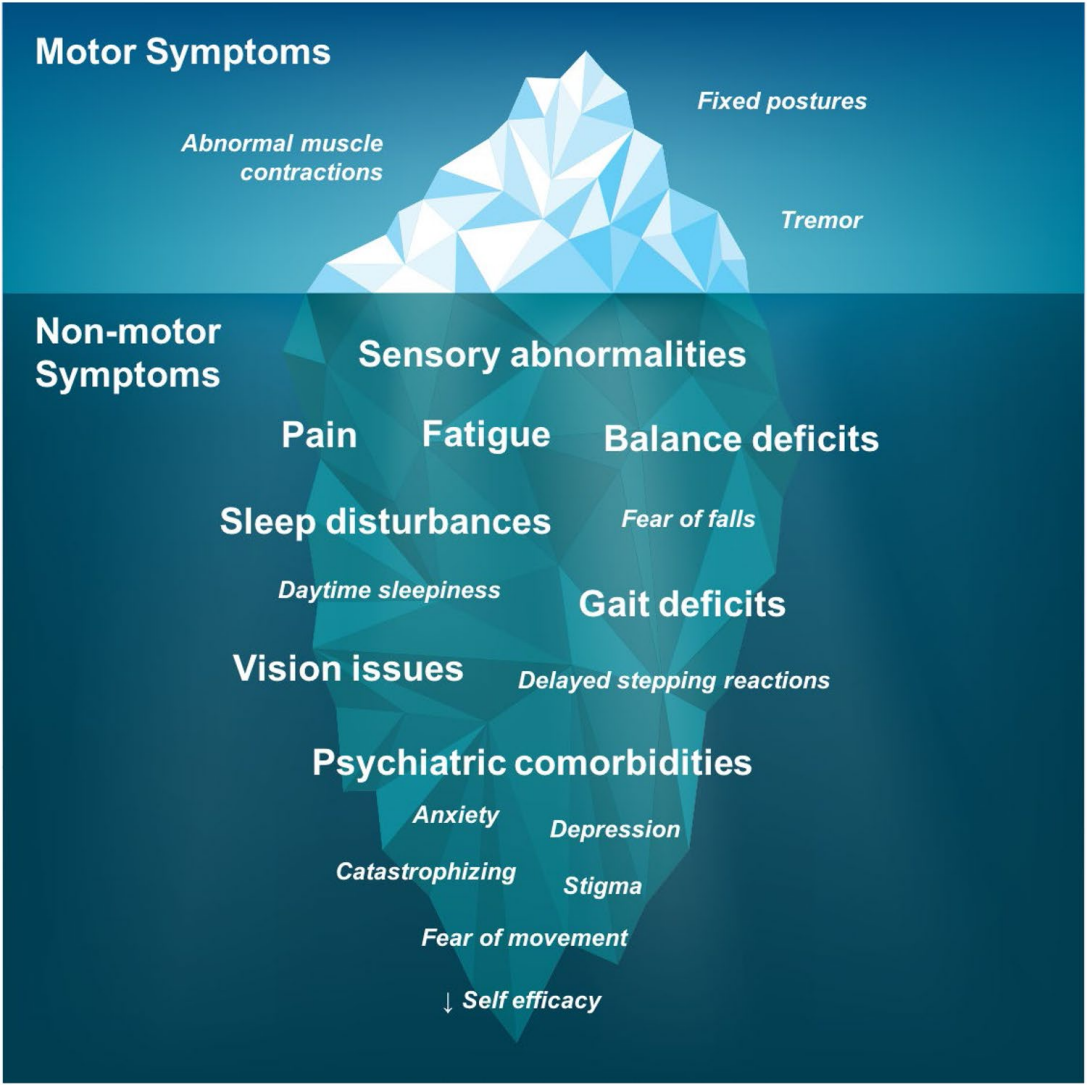
Visualisation of the motor and non-motor symptoms of dystonia as an ice-berg with physical symptoms that can be seen, but non-motor symptoms that often go unseen

Treatments for dystonia offer limited effectiveness and patient satisfaction. A reason for this could be that current therapies are too narrow focused and have not considered the wider impacts of dystonia on motor function as well as non-motor symptoms (Batla et al. 2019; Torres and Rosales 2017; Franco and Rosales 2015). In Fig. 4, we have depicted the motor and non-motor symptoms of dystonia on an ice-berg where the motor symptoms are often seen as the problem that needs to be addressed, but there are many more unseen problems that are significant contributors to a person’s level of disability and quality of life (Pauw et al. 2017; Dool et al. 2016; Smit et al. 2016, 2017b; Zetterberg et al. 2009; Tomic et al. 2016). Tremor and jerks, pain and fatigue, balance and gait, fear of falling, vision issues, poor sleep, anxiety and depression cause significant disability, negative impact on daily life, and reduce quality of life (Dool et al. 2016; Smit et al. 2016, 2017a, b; Zetterberg et al. 2009; Tomic et al. 2016). Therapists should begin to consider and integrate all of these aspects into a person’s rehabilitation. Recognition and rehabilitation of the wider spectrum of dystonic signs and symptoms could be key to improving treatment outcomes, life participation, and overall quality of life for people living with dystonia.

Without a curative treatment for dystonia, the focus for therapists must be on alleviating the symptoms which encompass more than the dystonic muscle contraction. To begin to provide a holistic treatment, therapists will need to perform assessments of these issues. Physical therapists should include gait and physical function and balance tests (e.g. TUG, Berg Balance scale), and clinical assessment of gait in their overall assessment of a patient. In addition, non-motor symptoms could be screened for using a validated non-motor symptom scale (Smit et al. 2017a; Klingelhoefer et al. 2019). The Dystonia Non-Motor Symptoms Questionnaire (Smit et al. 2017a) includes multiple domains, such as sleep, fatigue, emotional well-being, sensory symptoms, activities of daily living, autonomic symptoms and stigma, and could be easily implemented into routine clinical practice. The multitude of findings that people living with dystonia experience significant psychological issues, such as depression and anxiety, fear of falling and heightened feelings of stigma, strongly suggests that a multi-disciplinary approach to rehabilitation is needed. Self-efficacy and self-management strategies are vital for adherence to long-term rehabilitation programs. Self-management interventions are another component of treatment for dystonia that is currently under explored and should urgently be addressed.

Finally, an awareness of maintaining an overall healthy lifestyle is also needed for people with dystonia. In our self-reported PA research, we found that people were reporting adequate levels of PA, though they did not feel supported or guided with their exercise routines (McCambridge et al. 2019). We are currently following up this research with objective measurements, and we hope to better understand the health impacts of dystonia on obesity and other co-morbidities. Exercise guidelines developed specifically for dystonia, that are prescribed to the patient by a trained professional, are needed to reduce barriers to participation and facilitate dystonia patients to engage in PA without symptom exacerbation. Only after careful consideration of these issues can we understand the longer term benefits of exercise and PA on participation and quality of life in people living with dystonia.

While the practice of neurorehabilitation has its place in dystonia (Franco and Rosales 2015), a holistic rehabilitation approach is needed for managing the myriad of symptoms, both motor and non-motor, experienced by people living with isolated dystonia (Batla et al. 2019). Motor symptoms such sustained muscle contractions and abnormal postures, tremor and muscle jerks are often the main focus of treatment. However, pain, depression, stigma, impaired sleep, and fatigue are only a few of the debilitating non-motor symptoms that contribute to the lived experience of dystonia (Torres and Rosales 2017). Given that non-motor symptoms have been found to decrease health-related quality of life more so than motor symptoms (Smit et al. 2016, 2017a, b; Torres and Rosales 2017; Zetterberg et al. 2012; Wagle Shukla et al. 2016), a multi-disciplinary approach to rehabilitation must be adopted. Future studies should continue to explore the wider impairments associated with dystonia, and the impact of a holistic rehabilitation approach using carefully designed multi-disciplinary clinical trials on patient-reported outcome measures.
